# Double Trouble: A Case of Two Simultaneous Thrombotic Events in a Patient With COVID-19

**DOI:** 10.7759/cureus.24912

**Published:** 2022-05-11

**Authors:** Alexander Landsman, James R Pellegrini, Muhammad S Tiwana, Shivankshi Berry, Jaswinder Singh

**Affiliations:** 1 Internal Medicine, Nassau University Medical Center, East Meadow, USA

**Keywords:** thromboembolic disease, ischemic cerebrovascular disease, pulmonary embolism (pe), therapeutic anticoagulation, prophylactic anticoagulation, hypercoagulable state, covid-19

## Abstract

Coronavirus disease 2019 (COVID-19) is known to primarily have respiratory tract involvement, but thromboembolic complications can occur as well, leading to increased overall mortality seen in these patients. We present a case of a patient infected with COVID-19 who then developed two simultaneous thrombotic events.

Our patient is a 57-year-old male who presented to the emergency department with sudden onset dysarthria and left lower extremity weakness. Medical records indicated he recently tested positive for COVID-19 infection 10 days ago. Magnetic resonance imaging (MRI) of the brain revealed an acute right cerebellar infarction as well as acute bilateral thalamic infarcts. Later in the hospital course, computed tomography angiography (CTA) of the chest revealed a right lower lobe segmental pulmonary artery embolism.

Patients with COVID-19 have been seen to develop a wide spectrum of thromboembolic manifestations, most commonly being venous thromboembolism. Arterial thrombosis and microvascular disease can be detected as well. Early diagnosis and treatment of clotting disease is essential and may decrease overall mortality in COVID-19-infected patients.

## Introduction

A new coronavirus, severe acute respiratory syndrome coronavirus 2 (SARS-CoV-2), appeared in early 2019 and altered the path of history. Coronavirus disease 2019 (COVID-19) is known to primarily affect the respiratory tract with severe presentations manifesting as acute respiratory distress syndrome (ARDS), acute kidney injury (AKI), liver damage, and thromboembolic events as well [1]. When treating COVID-19 patients it is imperative that clinicians rule out thromboembolic disease in light of the hypercoagulable state COVID-19 is known to cause [2]. We present a fascinating case of a COVID-19-infected patient who developed multiple thromboembolic events.

## Case presentation

**Figure 1:**
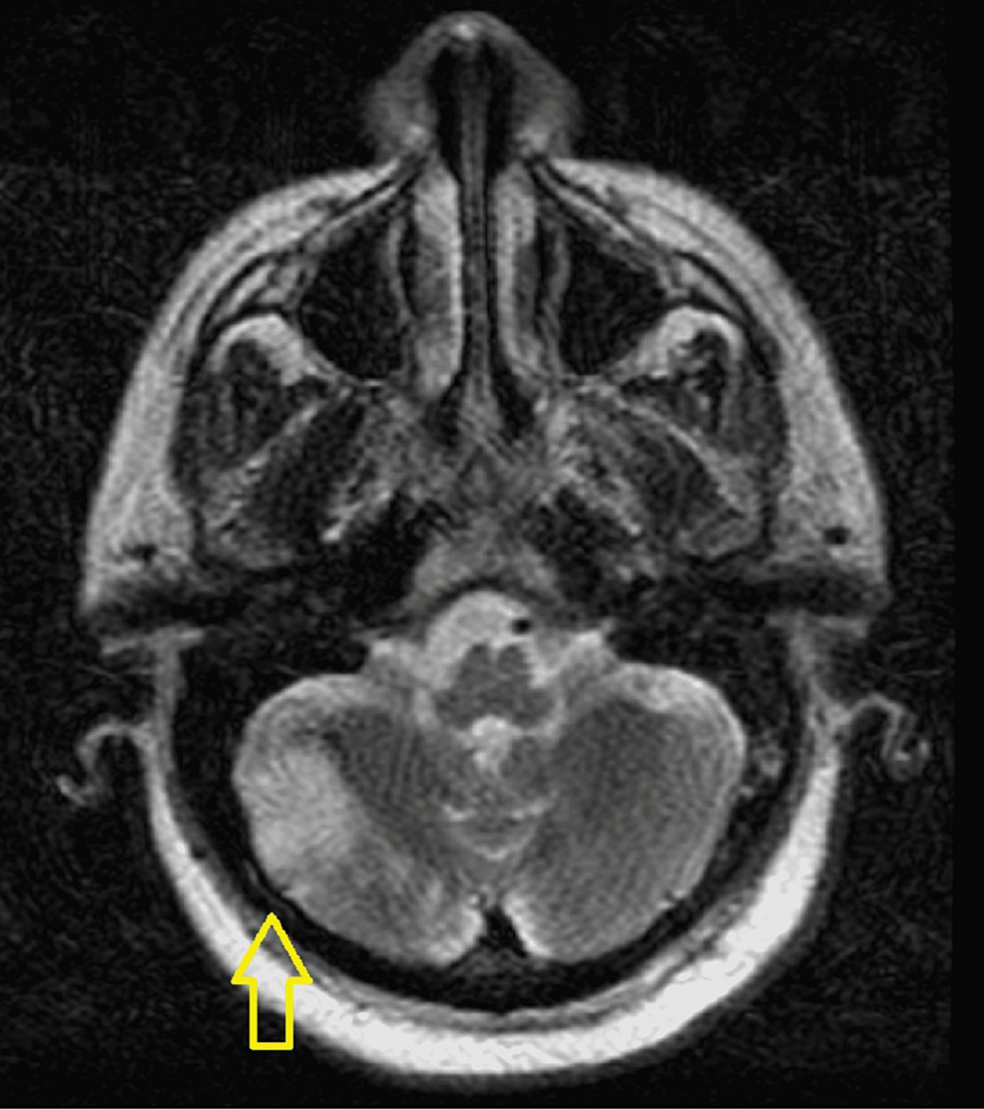
MRI of the brain showing right-sided cerebellar infarct.

A 57-year-old male with a past medical history of hypertension, diabetes mellitus type 2, and hyperlipidemia presented to the emergency department with sudden onset dysarthria and left lower extremity weakness. Prior medical records indicated he recently tested positive for COVID-19 infection 10 days ago. Upon further history, the patient revealed he had already received two doses of the Pfizer-BioNTech COVID-19 vaccine and denied any prior history of clotting disease. The patient’s last known well time was approximately nine hours prior to arrival. On physical examination, the patient had a National Institutes of Health Stroke Scale (NIHSS) score of 6 due to aphasia and left lower extremity drift. The patient’s blood pressure was 153/100 mmHg, heart rate 92 beats per minute, respiratory rate 18 breaths per minute, temperature 98.3°F, and oxygen saturation was 100% on room air. COVID-19 polymerase chain reaction (PCR) testing was positive. He denied any respiratory symptoms at that time. Computed tomography (CT) scan of the head without contrast revealed acute bilateral thalamic infarcts as well as a left posterior cerebral artery (PCA) occlusion, and no signs of hemorrhage. The patient was found to be outside of the time window period for tissue plasminogen activator (tPA) administration and was also deemed not a candidate for thrombectomy in light of the distal location of the PCA occlusion. MRI of the brain revealed an acute right cerebellar infarction as well as acute bilateral thalamic infarcts (Figure 1).

**Figure 2:**
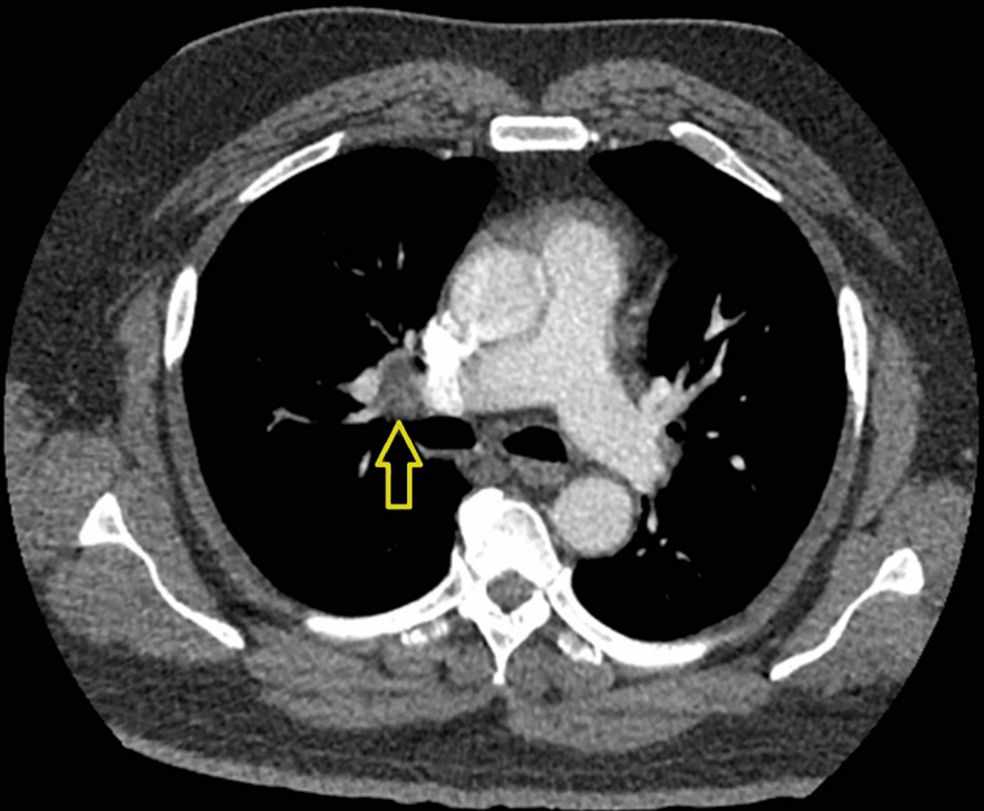
CT imaging showing a right lower lobe segmental pulmonary artery embolism.

The patient was admitted to the medical intensive care unit (MICU) and was initiated on aspirin, clopidogrel, high-intensity statin, and subcutaneous heparin for thromboembolic prophylaxis. On the third day of admission, the patient suddenly developed shortness of breath and tachycardia with no clinical or biochemical signs of hypoxia. D-dimer level was elevated at 0.98 μg/mL. C-reactive protein and fibrinogen levels were abnormally high as well. An urgent CTA of the chest revealed a right lower lobe segmental pulmonary artery embolism (Figure 2). Bilateral lower extremity Doppler ultrasound revealed no evidence of deep vein thrombosis (DVT). The patient was started on oral apixaban for the treatment of pulmonary embolism (PE). He steadily improved clinically with resolution of dyspnea and tachycardia. Aggressive physical rehabilitation allowed for improvement in his neurologic deficits. The patient was eventually discharged and given outpatient appointments for follow-up.

## Discussion

COVID-19 has been shown to have increased overall risk for thromboembolic events which may be an added factor to the high mortality rate seen in these patients. One meta-analysis comprising more than 40 studies and close to 18,000 patients shows a pooled incidence of venous thromboembolic events (VTE) in patients with COVID-19 to be 17% (12% for DVT and 7.1% for PE) [3]. Another study analyzing over 3,000 COVID-19 patients detected an overall incidence of 16.5% for PE and 14.8% for DVT [4]. Accurate assessments of the true incidence of VTE in hospitalized patients with COVID-19 remain elusive, with estimates ranging from 4.8% to 85% [3]. The presence of VTE in hospitalized patients with COVID-19 is associated with greater disease severity and increased mortality. Patients with PE more frequently require mechanical ventilation and ICU admission and have increased overall hospital length of stay. In another study including 184 patients with severe COVID-19 infection, VTE incidence was reported at 27% with an overall 13% mortality rate [5].

The association of thromboembolic disease with the COVID-19 infection can be understood through the lens of the three components of Virchow’s triad. Direct invasion of endothelial cells by the SARS-CoV-2 virus points to the first element of the triad, endothelial injury. Studies have demonstrated the presence of actual viral particles inside endothelial cells [6,7]. Cytokine release as well as increased levels of acute-phase reactants can also contribute to the endothelial breakdown. The second component is stasis of blood, as seen in hospitalized patients with prolonged periods of immobilization. The third component, hypercoagulable state, is attributed to the elevated numbers of circulating prothrombotic factors including von Willebrand factor (vWF), factor VIII, D-dimer, and fibrinogen [8]. High D-dimer levels in COVID-19 patients have been correlated with worsening disease progression as well as increased risk for thrombotic events [9].

Patients with COVID-19 have been seen to develop a wide spectrum of thromboembolic manifestations, most commonly being venous thromboembolism, including DVT and PE. Arterial thromboses such as ischemic stroke, limb ischemia, and myocardial infarction have also been reported. Autopsy reports and lung biopsies have shown evidence of microvascular thrombotic injury [10]. Interestingly, our patient developed both venous and arterial thromboses in the form of venous PE and arterial cerebellar infarct.

Current guidelines suggest patients admitted to the hospital with COVID-19 infection should be evaluated with routine blood testing including complete blood count (CBC) and coagulation studies such as prothrombin time (PT), partial thromboplastin time (PTT), fibrinogen, and D-dimer. Laboratory blood testing can be trended daily or less frequently depending on disease severity and clinical judgment. Abnormal coagulation values can offer prognostic value. Typically, COVID-19 patients can have high D-dimer and fibrinogen levels which are correlated with a poorer prognosis. Routine imaging to assess for clotting disease is not recommended, however, the threshold for evaluation of DVT or PE should be low in these patients. The American Society of Hematology (ASH) suggests for any patient with suspected PE due to unexplained symptoms of worsening respiratory status, hypotension, or tachycardia computed tomography with pulmonary angiography (CTPA) is the preferred test to confirm or exclude the diagnosis. Further imaging should be dependent on clinical evaluation and should be patient-specific [11].

Up until January 2022, both the National Institute of Health (NIH) and ASH had recommended prophylactic dose only as anticoagulation for COVID-19 patients [12,13]. A recent New England Journal of Medicine article published in August 2021 argues for the use of therapeutic dose of heparin for anticoagulation in non-critically ill hospitalized patients infected with COVID-19 [14]. The article concluded that the group receiving the therapeutic dose of heparin had increased overall survival compared to the group receiving the prophylactic dose. Based on these findings the NIH currently recommends using therapeutic doses of heparin for COVID-19-infected patients who are hospitalized and require low-flow oxygen. The patient discussed in our report never required supplemental oxygen and therefore was not treated initially with a therapeutic dose of anticoagulation.

Based on the current clinical research and case reports such as ours, the guidelines for anticoagulation in patients infected with COVID-19 are dynamic and constantly under review. We suggest that this case report can possibly be evidence to argue that prophylactic dose of subcutaneous heparin was insufficient to prevent thrombotic events. Therapeutic dose of heparin may have prevented further thrombotic events from occurring and may increase overall morbidity and mortality in COVID-19-infected patients, even those who do not require supplemental oxygen. A further review, however, is needed to establish a precise underlying etiology of the thrombotic events that occurred in our patient. Underlying medical conditions such as diabetes and hypertension may have predisposed the patient to ischemic strokes. Hospitalization and decreased mobility may have led to the development of PE.

## Conclusions

Any patient hospitalized with COVID-19 infection should warrant a high clinical suspicion for underlying thromboembolic complications. Early diagnosis and proper treatment of these complications is critical and may improve overall mortality related to COVID-19. Guidelines to use anticoagulants in patients infected with COVID-19 remain dynamic and are constantly under review. Our case report argues that a prophylactic dose of subcutaneous heparin is insufficient to prevent thrombotic events in COVID-19 patients. Therapeutic dose of heparin may have prevented further thrombotic events from occurring in our patient and may increase overall morbidity and mortality in COVID-19-infected patients. Further studies and rigorous analysis of treatment guidelines are warranted to better understand the underlying etiology of thrombosis in COVID-19 patients and prevent related thrombotic disease.
